# The role of BRAF V600 mutation in melanoma

**DOI:** 10.1186/1479-5876-10-85

**Published:** 2012-07-09

**Authors:** Paolo A Ascierto, John M Kirkwood, Jean-Jacques Grob, Ester Simeone, Antonio M Grimaldi, Michele Maio, Giuseppe Palmieri, Alessandro Testori, Francesco M Marincola, Nicola Mozzillo

**Affiliations:** 1 Department of Melanoma, Istituto Nazionale Tumori Fondazione “G. Pascale”, Naples, Italy; 2 Department of Medicine, Division of Hematology/Oncology, University of Pittsburgh Cancer Institute, Pittsburgh, PA, USA; 3 Hopital de la Timone, and Aix-Marseille Univ, 264 Rue St Pierre, 13885, Marseille CEDEX 05, Marseille, France; 4 Medical Oncology and Immunotherapy, Department. of Oncology, University Hospital of of Siena, Istituto Toscano Tumori, Siena, Italy; 5 Unit of Cancer Genetics, Institute of Biomolecular Chemistry, National Research Council (CNR), Sassari, Italy; 6 Melanoma and muscle-cutaneous sarcomas Division, Istituto Europeo di Oncologia, Milan, Italy; 7 Infectious Disease and Immunogenetics Section (IDIS), Department of Transfusion Medicine, Clinical Center and Center for Human Immunology (CHI), NIH, Bethesda, MD, USA; 8 Unit of Medical Oncology and Innovative Therapy, Istituto Nazionale per lo Studio e la Cura dei Tumori “Fondazione G. Pascale”, Via Mariano Semmola, 80131, Naples, Italy

**Keywords:** BRAF, Vemurafenib, Melanoma

## Abstract

BRAF is a serine/threonine protein kinase activating the MAP kinase/ERK-signaling pathway. About 50 % of melanomas harbors activating BRAF mutations (over 90 % V600E). BRAFV600E has been implicated in different mechanisms underlying melanomagenesis, most of which due to the deregulated activation of the downstream MEK/ERK effectors. The first selective inhibitor of mutant BRAF, vemurafenib, after highly encouraging results of the phase I and II trial, was compared to dacarbazine in a phase III trial in treatment-naïve patients (BRIM-3). The study results showed a relative reduction of 63 % in risk of death and 74 % in risk of tumor progression. Considering all trials so far completed, median overall survival reached approximately 16 months for vemurafenib compared to less than 10 months for dacarbazine treatment. Vemurafenib has been extensively tested on melanoma patients expressing the BRAFV600E mutated form; it has been demonstrated to be also effective in inhibiting melanomas carrying the V600K mutation. In 2011, both FDA and EMA therefore approved vemurafenib for metastatic melanoma carrying BRAFV600 mutations. Some findings suggest that continuation of vemurafenib treatment is potentially beneficial after local therapy in a subset of patients with disease progression (PD). Among who continued vemurafenib >30 days after local therapy of PD lesion(s), a median overall survival was not reached, with a median follow-up of 15.5 months from initiation of BRAF inhibitor therapy. For patients who did not continue treatment, median overall survival from the time of disease progression was 1.4 months. A clinical phase I/II trial is evaluating the safety, tolerability and efficacy of vemurafenib in combination with the CTLA-4 inhibitor mAb ipilimumab. In the BRIM-7 trial vemurafenib is tested in association with GDC-0973, a potent and highly selective inhibitor of MEK1/2. Preliminary data seem to indicate that an additional inhibitor of mutated BRAF, GSK2118436, might be also active on a wider range of BRAF mutations (V600E-K-D-R); actually, treatment with such a compound is under evaluation in a phase III study among stage III-IV melanoma patients positive for BRAF mutations. Overall, BRAF inhibitors were well tolerated; common adverse events are arthralgia, rash, fatigue, alopecia, keratoacanthoma or cutaneous squamous-cell carcinoma, photosensitivity, nausea, and diarrhea, with some variants between different inhibitors.

## Introduction

BRAF is a serine/threonine protein kinase, encoded on chromosome 7q34, that activates the MAP kinase/ERK-signaling pathway. BRAF is the family member most easily activated by Ras [1,2]. In addition, the basal kinase activity of BRAF is higher than that of other family members [3,4]. This provides a potential rationale for the frequent mutational activation of BRAF observed in human tumors [5].

In fact, approximately 50 % of melanomas harbor activating BRAF mutations. Among the BRAF mutations observed in melanoma, over 90 % are at codon 600, and among these, over 90 % are a single nucleotide mutation resulting in substitution of glutamic acid for valine (BRAFV600E: nucleotide 1799 T > A; codon GTG > GAG). The second most common mutation is BRAFV600K substituting lysine for valine, that represents 5-6 % (GTG > AAG), followed by BRAFV600R (GTG > AGG), an infrequent two-nucleotide variation of the predominant mutation, BRAF V600 ′E2′ (GTG > GAA), and BRAF V600D (GTG > GAT) [6]. The prevalence of BRAFV600K has been reported as higher in some populations [7].

In melanoma, BRAF mutation is most common in patients whose tumors arise on skin without chronic sun-induced damage, whereas BRAF mutations are rare in melanomas arising from mucosal and acral sites [8].

A major advance of the past few years was the discovery that RAF kinases can homo- and heterodimerize [9,10], and that, in fact, the structure of an active RAF kinase is that of a side-to-side dimer in which only one partner must have catalytic activity [11]. Dimerization is enhanced by Ras [12] and is subject to negative feedback regulation by ERK [10,13].

Several RAF mutations have been implicated in the induction of genomic instability, driving the proliferation of cancer cells with the highest frequency in melanoma. For instance, mutated BRAF signals as a monomer, independent of upstream growth stimuli. The most frequent BRAF mutation, BRAFV600E, causes constitutive activation of the kinase as well as insensitivity to negative feedback mechanisms [5,14].

BRAFV600E has been implicated in different mechanisms of melanoma progression, and principally the activation of the downstream MEK/ERK pathway, evasion of senescence and apoptosis, unchecked replicative potential, angiogenesis (through MEK-dependent activation of HIF-1α and VEGF), tissue invasion and metastasis (via upregulation of several proteins involved in migration, integrin signaling, cell contractility, tumor- and microenvironment-derived interleukin-8), as well as the evasion of immune response [15].

No clear differences in prognosis (time from primary diagnosis to distant metastasis) were noted between BRAF-mutated versus wild-type melanomas. Features of the antecedent primary melanoma significantly associated with a BRAF mutation (*P* < 0.05) were the superficial spreading and nodular histopathological subtypes, the presence of mitoses, the presence of a single or occult primary melanoma, a truncal location and age at diagnosis of the primary tumor (≤50 years) [7].

**Figure 1:**
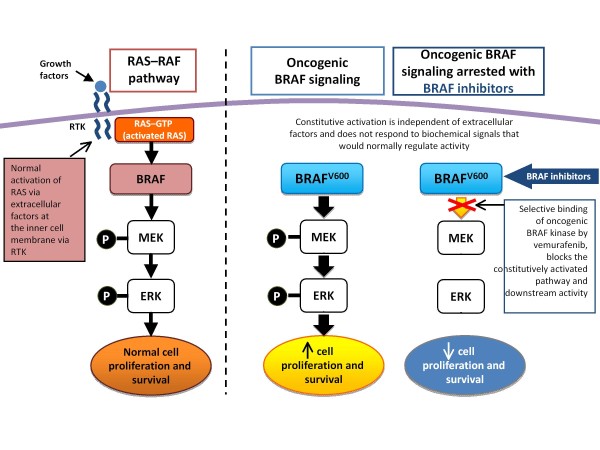
Oncogenic BRAF signaling pathway. RTK: receptor tyrosine kinase.

The discovery of the genetic underpinnings of melanoma and their characterization have exposed potential targets for therapy, BRAF mutations being principal among them (Figure 1) [16].

### BRAF inhibition: previous experience

Sorafenib, a non-selective BRAF broad-spectrum kinase inhibitor with a bi-aryl urea structure, was originally developed in combination with carboplatin and taxol against lung cancer. Activity against melanoma was demonstrated in phase I studies, and so it was further developed for this indication in combination with the unusual combination of carboplatin and paclitaxel. Because of response rates over 30 % in phase II studies, its development was pursued into large phase III trials, but eventually it failed both in second- and first-line trials [17]. In fact, the addition of sorafenib to carboplatin and paclitaxel (CP) did not improve any of the relevant end points over placebo plus CP in the second-line setting for patients with advanced melanoma [18].

Studies of sorafenib indicate that it lacks selectivity and potency for RAF, and that it is a highly potent inhibitor of VEGFR2, VEGFR3, and several other kinases [19]. Sorafenib ultimately demonstrated clinical activity in renal cell carcinoma (RCC) and hepatocellular cancer and is now approved for use in these indications. These findings suggest that the activity of sorafenib in RCC is likely attributable to its anti-angiogenic properties and that inhibition of RAF contributes little if at all to its clinical efficacy in this disease.

### Vemurafenib: phase I and II results

In phase III studies, dacarbazine, the only chemotherapeutic agent approved by the U.S. Food and Drug Administration (FDA) for the treatment of metastatic melanoma, was associated with a response rate of 7 to 12 % and a median overall survival of 5.6 to 7.8 months after the initiation of treatment.

**Figure 2:**
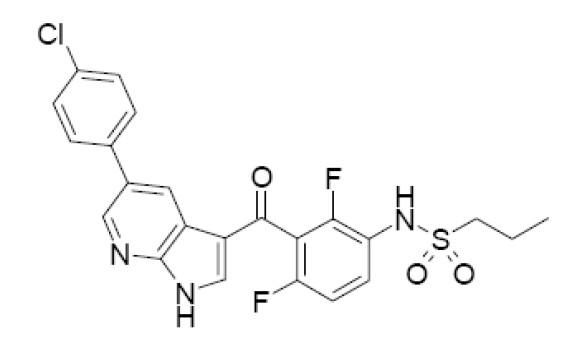
The structural formula of vemurafenib.

Recently a selective and potent inhibitor of oncogenic mutant BRAF [20], vemurafenib (PLX4032/RG7204/RO5185426), gave positive results in phase I [21] and phase II trials (Figure 2) [22].

The phase I study was a multicenter, 55-solid cancer patients dose-escalation trial followed by a 32-melanoma patients extension phase involving the maximum dose that could be administered without adverse effects (the recommended phase II dose). Vemurafenib was administered at a starting dose of 160 mg daily and was generally well tolerated with no dose-limiting toxicity until the 720 mg twice-daily dose level was initiated. This trial demonstrated that vemurafenib has very impressive single-agent clinical activity, with unprecedented objective response rates (complete plus partial tumor regression) in about 81 % and a confirmed response rate in about 56%of patients who had melanoma with the BRAFV600E mutation; the study also showed a clear impact on PFS >7 months and established the maximum tolerated dose to be 960 mg twice-daily. Responses were observed at all sites of disease, including the bone, liver and small bowel. During the dose escalation phase, responses were also observed in patients who were receiving doses below the recommended one [21].

The phase II trial involving patients who had received previous treatment for melanoma with the BRAFV600E mutation investigated the efficacy of vemurafenib with respect to overall response rate (primary end-point, defined as percentage of treated patients with a tumor response), duration of response and overall survival. The study enrolled 132 patients who were administered vemurafenib at a dose of 960 mg orally twice-daily (until the development of unacceptable toxic effects or disease progression) and showed a confirmed response rate of 53 %, with a median duration of response of 6.7 months and a median overall survival of 15.9 months, an unprecedented outcome in melanoma patients.

The overall survival rate at 6 months was 77 % (95 % CI, 70 to 85), 58 % at 12 months (95 % CI, 49 to 67) and estimated to be 43 % at 18 months (95 % CI, 33 to 53). During the follow-up period (median was 12.9 months, range 0.6 to 20.1), 24 % patients received ipilimumab after they had disease progression while receiving vemurafenib. In an unplanned post hoc analysis, median overall survival remained at 15.9 months (95 % CI, 8.0 to not reached) even when these ipilimumab-treated patients were not included [22].

### Vemurafenib: the BRIM-3 results

The 2-arm randomized phase III trial in treatment-naïve patients (BRIM-3) compared vemurafenib, 960 mg orally twice-daily, to dacarbazine chemotherapy, 1,000 mg/m^2^ administered every 3 weeks. Progression-free survival (PFS) and overall survival were co-primary end-points in this 675 patients-trial on unresectable, previously untreated stage IIIC or stage IV metastatic melanoma with the BRAFV600E mutation. The study results were associated with a relative reduction of 63 % in the risk of death and of 74 % in the risk of tumor progression in patients. To date, after a longer follow-up (data cut-off 3^rd^ October 2011) the median OS for vemurafenib arm is of 13.2 months compared to 9.9 months in the dacarbazine arm [23]. Notably, 38 % of patients required dose reduction in the vemurafenib arm.

Common adverse events associated with vemurafenib were arthralgia, rash, fatigue, alopecia, keratoacanthoma or cutaneous squamous-cell carcinoma (cSCC), photosensitivity, nausea and diarrhea.

Vemurafenib was the first personalized compound which demonstrated an improvement in PFS and OS in metastatic melanoma harboring the BRAF V600 mutation [24].

### U.S. and European approval

On August 17th, 2011, the U.S. Food and Drug Administration (FDA) approved vemurafenib tablets for unresectable or metastatic melanoma with the BRAFV600E mutation as detected by a concurrently FDA-approved test. The FDA has stamped an early approval on vemurafenib two months ahead of its PDUFA date. It was in fact reviewed under the FDA’s priority review program that provides for an expedited six-month review of drugs that may offer major advances in treatment or that provide a treatment when no adequate therapy exists.

On December 15th, 2011, the European Medicine Agency (EMA)’s Committee for Human Medicinal Products (CHMP) recommended the granting of a marketing authorization for vemurafenib to treat patients with metastatic or unresectable melanoma and the BRAFV600 mutation. European Commission approval for marketing in 27 EU countries was finally granted on February 17th, 2012.

Despite this early approval for European marketing, it is noteworthy that there exist important differences in market access throughout the 27 EU countries. In fact, each country needs additional time to obtain reimbursement from its own national drug agency, which depends on more or less structured internal processes.

Recent data show that there is a broad range of accessibility times: in particular, the Patients W.A.I.T. (Waiting to Access Innovative Therapies) Indicator 2010 Report for new medicines in the period between 1 January 2007 and 31 December 2009 based on EFPIA’s database shows that average time elapsing between the date of first valid EU market authorization and the accessibility date (i.e. date of completion of pricing/reimbursement procedures) in 14 European countries varies from 88 (Austria) to 392 days (Belgium) (not considering Germany and the UK). In Italy this time is of 326 days [25].

### The mutated-BRAF approach

In melanoma, vemurafenib represents the first drug of a lineage exerting its antiproliferative activity through inhibition of a highly specific molecular target. First results with other targeted BRAF inhibitors like dabrafenib (GSK2118436) have provided similar dramatic results, with a similar profile of toxicity, thus comforting both relevance and robustness of the approach aimed at inhibiting the mutated BRAF.

In particular, the response rates reported in a phase I study with dabrafenib was about 60 % in melanoma patients carrying the BRAF-V600E/K mutation [26]. The phase II study showed an overall response rate of 59 % and a PFS of 27.4 weeks in the BRAF-V600E mutated population [27]. A Phase III study comparing the activity of dabrafenib with dacarbazine is currently ongoing: the sample population will include 200 BRAF-V600E mutated patients and the primary endpoint will be the PFS, since the study will allow crossover (NCT01227889).

RAF265 is a BRAF-V600E and VEGFR2 inhibitor that showed dose-dependent inhibition of tumor growth and tumor regression in xenografts presenting BRAF-V600E mutant cells. The first clinical experience was reported in 2011. In a phase I study of 71 evaluable patients response rates were disappointing: 16 % in patients with BRAF mutations and 13 % in patients with wild-type BRAF [28].

LGX818 in another BRAF inhibitor selective for the BRAF-V600E mutation which is under phase I investigation (NCT01436656).

### BRAFV600 mutation test: who should do this?

A BRAFV600 mutation test is necessary to determine if a patient might be a candidate for vemurafenib therapy. Together with the BRAF inhibitor, a mutation test has been approved by FDA, which is able to detect V600E, V600K and V600D substitutions more sensitively than Sanger sequencing [29]. In many countries, strategies dedicated to tumor typing and based on different techniques have been settled. In any case quality control is warranted.

Taking into account the evidence from clinical trials, we strongly support the need to screen for the BRAF-V600 mutation all patients with advanced melanoma (unresectable stage III and stage IV), who are most likely to derive benefit from vemurafenib treatment, especially when symptomatic with their disease. Patients with a high risk for recurrence (stage IIIB and IIIC) are also reasonable to consider for mutation screening.

The adjuvant role of this agent is being studied in the BRIM-8 trial. This is a phase III, randomized, double-blind, placebo-controlled study of vemurafenib adjuvant therapy in patients with surgically-resected cutaneous BRAF mutant melanoma at high risk for recurrence.

### The role of vemurafenib and ipilimumab in the treatment of advanced melanoma

The actual place of the novel anti-CTLA-4 mAb ipilimumab [30,31] is obvious in non mutant BRAF patients, but is still a matter in BRAF mutant patients which has shown an impact directly on OS. Ipilimumab has a very different profile from BRAF-inhibitors with a slow action and an impact directly on OS, often without evidence of immediate response. In clinical practice, it may seem relevant to initially treat asymptomatic advanced stage IV M1a/b BRAF mutant melanoma patients with high-dose IL-2 or ipilimumab. The reasons for this lie in the mechanisms of action of immunotherapy and the singular durable benefit that is recorded with mature trials of both of these immunotherapy agents, which (a) require a period of 1.5-3 months for assessment, and (b) have a durable impact on OS often without an acute influence upon tumor size and symptoms. However, the median OS reached in phase II (15.9 months) and the phase III study (13.2 months) with vemurafenib represent the most favorable impact upon OS seen in melanoma trials to date. The setting of metastatic melanoma and in particular those patients with low tumor burden has been recently studied by Amaravadi et al [32]. They showed that the median survival of Phase I BRAF V600E melanoma patients treated with vemurafenib was 16 months, and the 54 % and 44 % of patients were still alive at 1 and 2 years, respectively. The recent results of BRIM2 study [22] are consonant with this. Most important, patients with >12 months’ PFS had a significantly lower baseline tumor burden compared with patients with PFS <6 months, evaluated using RECIST. In fact, considering a baseline target lesion cut-off value of 11.5 cm of diameter, in patients with a target tumor burden < 11.5 cm the median PFS and OS was of 16 and 27.1 months, respectively; while, in patients with a target > 11.5 cm the PFS and OS was of 6.6 and 12.0 months, respectively [32]. This finding suggests an impact of vemurafenib in the patients with a low tumor burden. This may provide part of the rationale for the evaluation of vemurafenib and BRAF inhibitors in metastatic melanoma apart from symptomatic patients, as well as for consideration of this agent in the adjuvant setting, as a single agent.

### B-raf inhibitors: pathways involved in drug resistance

Even if the majority of patients treated with vemurafenib show a shrinkage of tumor lesions, soon after the first evidence of objective response was observed with vemurafenib, evidence of disease progression quickly manifested in some patients. The range of response duration with this therapy is quite broad [33].

There appear to be both MAPK pathway-dependent and MAPK pathway-independent mechanisms by which tumors can survive and adapt in the setting of BRAF inhibitor therapy. In the majority of cases, there is biochemical evidence of reactivation of the MAPK pathway: the appearance of concomitant NRAS mutations; the appearance of a MEK mutation has been described; increased expression of COT kinase [34].

Additional mechanisms appear to be predominantly activated by signaling to the PI3K pathway. These include upregulation of platelet-derived growth factor (PDGF) receptor and insulin-like growth factor (IGF) receptor [35].

Considering these evidences of disease progression after vemurafenib treatment, clinical trials have already started to test combination or succession of therapies in melanoma patients with high risk for recurrence or who have progressed [36,37]. For instance, combination with a MEK inhibitor has been hypothesized to prolong PFS and appears to prevent the emergence of skin toxicities on the basis of experimental and first clinical results.

### B-raf inhibitors beyond progression

For a subset of patients with disease progression, continuation of vemurafenib treatment is potentially beneficial after local therapy (surgery or radiotherapy). Kim et al. pointed out pattern and outcome of disease progression from the phase I vemurafenib clinical trial [33]. Common sites of disease progression were: skin/soft tissue (44 % of all 48 pts); nodes (27 %); brain/CNS (25 %); lungs (19 %); liver (15 %); bone, GI (10 %). Among 42 pts with PD, 19 (45 %) progressed only in new sites, 8 (19 %) in the brain only and 11 (26 %) in both new and original sites. Among 18 pts who continued vemurafenib >30 days after a local alternative therapy of a site of disease progression, a median overall survival exceeds 15.5 months from initiation of BRAF inhibitor therapy. Median treatment duration beyond initial disease progression was 3.6 months (range, 1.1–9.9) and median overall survival from the time of initial disease progression was not reached (median follow-up 6.0 months). For patients who did not continue treatment, median overall survival from the time of disease progression was 1.4 months. Adverse events during continued dosing in these patients were similar to those observed before disease progression [33]. Obviously, these results require prospective assessment in trials where additional therapies are added in proscribed time-frames.

### B-raf inhibitors in combination trials

A clinical phase I/II trial has been designed to evaluate the safety, tolerability, and efficacy of vemurafenib in combination with the Cytotoxic T Lymphocyte Antigen 4 (CTLA-4) inhibitor mAb ipilimumab [37]. This kind of combined treatment merges target therapy and immunotherapy approaches. The two drugs show different mechanisms of action, with ipilimumab (first- and second-line treatment in USA, second-line treatment in Europe to treat patients with late-stage metastatic unresectable melanoma) sustaining an active immune response. B-Raf inhibitors and ipilimumab also show a different pattern of action: while vemurafenib has been demonstrated to have quick action, rapid metabolic shutdown, but disease progression after a median of 6–8 months, the mAb shares a slow action with the ability to make the disease chronic.

Another kind of approach is being tested in the BRIM-7 trial [37]. This phase Ib, dose-escalation study aims at evaluating the safety, tolerability and pharmacokinetics of vemurafenib in combination with GDC-0973 in patients with BRAFV600E positive metastatic melanoma who have progressed after treatment with vemurafenib alone. GDC-0973 is a potent and highly selective inhibitor of MEK1/2, downstream targets of BRAF. The rationale underlying this combined therapy is two-fold: the first is the expectation of additive or possibly synergistic effects upon PFS; the second concerns the possibility avoidance of toxicities that may accompany the stimulation of the MEK pathway when BRAF inhibitors are used as single agents. This may prove to be a basis for the cSCCs and keratoacanthomas reported in most trials of BRAF inhibitors alone. Co-treatment with a MEK inhibitor is a rational approach to attenuating the skin toxicities of RAF inhibitor treatment and may also enhance the antitumor effects of RAF inhibitors by synergistically suppressing ERK pathway activity [38]. Recent phase I and II trials showed that this kind of approach is safe and able to lower the toxicity of either agent alone [39].

### BRAF inhibitor toxicities

Vemurafenib was well tolerated in all the clinical trials so far completed. In the BRIM-3 trial, the incidence of grade 1 to 2 and grade 3 to 4 adverse events was similar to those from prior studies. Common toxicities observed with vemurafenib include arthralgia, photosensitivity, rash, pruritus, alopecia, nausea, diarrhea and fatigue [24].

Sixty-one patients (18 %) had development of cSCC or keratoacanthoma. cSCCs and keratoacanthomas have previously been detected in patients treated with sorafenib, indicating a common mechanism in developing these adverse events. No metastatic evolution of RAF inhibitor-induced cSCCs has ever been reported.

The appearance of keratoacanthomas and cSCCs early in the course of treatment is speculated to involve the activating effect of vemurafenib on preneoplastic cells in which wild-type BRAF is further primed by upstream pathway activation. However, all these observed skin toxicities showed low metastatic potential, often regress spontaneously and are easily cured by surgical resection and/or destructive methods (cryotherapy or electrodessication/curettage) [38]. Several investigators have shown that vemurafenib and other inhibitors of RAF kinases can potentiate the activity of the MAPK pathway in cells with wild-type BRAF [40-42].

Dabrafenib has a similar safety profile of the vemurafenib [27]. However, vemurafenib was typically associated with photosensitivity (in approximately 30 % of treated patients BRIM3), whereas pyrexia (24 %) was mainly reported in dabrafenib treatment.

There is a concern about potential induction of new primary melanomas using BRAF inhibitors, but so far this risk is easily manageable in patients under surveillance, and has a low weight into the treatment of the metastatic disease.

### BRAF inhibitors: possible role in the BRAF-V600K patient population?

Even if vemurafenib has been extensively tested on melanoma patients expressing the BRAFV600E mutated form, it has also been demonstrated to be effective in inhibiting the V600K mutated form.

In fact, *in vitro* studies on melanoma cells isolated from primary or metastatic lesions showed that vemurafenib was also able to suppress the V600KBRAF activity [43]. Beside preclinical data demonstrating similar kinase activity of the V600K and V600E mutations, clear evidences of clinical activity of vemurafenib in patients with documented V600K mutation suggest that these patients are eligible to vemurafenib treatment too [44].

On behalf of the latter evidences, it is noteworthy that EMA’s CHMP positive opinion was not restricted to the V600E mutations like FDA approval, but included all kind of V600 mutations, comprising the less frequent ones.

In this sense, dabrafenib was also given for treatment of BRAF-V600K mutated patient (n = 16) in the phase II study [27], with an overall response rate of 13 % (and another 44 % with SD) and a PFS of 19.7 weeks, which demonstrated an impact even in this population.

### The role of BRAF inhibitors in brain metastases

Brain metastases (BM) are the most frequent intracranial tumors in adults and are up to ten fold more common than primary brain neoplasms. They are manifestations/complications of systemic tumors and in contrast to primary brain tumors do not constitute a separate disease entity [45]. Melanoma is the third most frequent primary tumor type in terms of brain metastasis, after lung and renal cell cancers [46]. BM are diagnosed in up to 10 % of melanoma patients during their disease course and BM are found at autopsy in up to 73 % of patients who died from disseminated cutaneous melanoma [47].

Patients with active BM have been excluded from prior and current vemurafenib trials. However, there are favorable preliminary efficacy data on other inhibitors of mutant BRAF, in patients with brain-metastatic melanoma [48] and a single-arm, phase II, multicenter study, evaluating efficacy and safety of vemurafenib in patients with brain-metastatic melanoma has been initiated (NCT01378975) [49]. Such systemic approaches are very promising, as expression of the therapeutic target (BRAF V600E-mutant protein) has been shown to be homogenous throughout the tumor tissue and to be consistent between different tumor manifestations in individual patients [50].

Analogously, dabrafenib showed good efficacy on brain metastases [26,51].

## Conclusions

Melanoma has historically had a poor prognosis because of lack of responsiveness to traditional chemotherapeutics as far as the finding that around one half harbors an activating mutation in BRAF leads to a challenging but promising focus for the development of novel targeted therapy.

This approach proved to be favorable since the first preclinic studies, whose results were then confirmed in clinical trials: vemurafenib represents an excellent model of anticancer targeted therapy, showing both unprecedented clinical activity and a good safety profile.

A diagnostic test to identify mutant BRAF melanoma patients that can receive benefit from vemurafenib treatment makes vemurafenib the first personalized targeted therapy in metastatic melanoma, able to recognize patients for whom treatment will more likely than not improve progression free and overall survival outcomes, with a tolerable safety profile.

Other drugs are currently under development and evaluation with the same target, like dabrafenib, or additional targets into the downstream pathway, and the results strongly confirm the concept firstly demonstrated for vemurafenib. As a consequence, future improvements of this targeted and personalized approaches are expected from ongoing clinical trials aiming at potentiate the activity of BRAF inhibitors through combination with other molecules, both immune-based and targeting the downstream pathway. These combination therapies also aim at lowering the observed skin toxicities.

## Competing interests

PAA participated to Advisory Board from Bristol Myers Squibb, MSD, Roche-Genentech, GSK, and received honoraria from Brystol Myers Squibb, MSD and Roche-Genentech. JMK has participated in advisory boards for Novartis and MSD, and receives honoraria as a consultant to GSK; he receives speaker honoraria from MSD. JJG participated to Advisory Board and thus received honoraria from Bristol Myers Squibb, MSD, Roche-Genentech, and GSK ES received honoraria from Bristol Myers Squibb. AMG has no Competing interest. MM Advisory boards from BMS and Roche, steering committee for GSK. GP has no Competing interest. AT has a consultant or advisory relationship with Bristol Myers Squibb, Amgen, Glaxo Smith-Kline, and received honoraria from them. Moreover, he received travel expenses refunded by Oncovision and IGEA. FMM has no Competing interest. NM has no Competing interest.

## Authors’ contributions

PAA, ES, AMG performed data acquisition, data analysis, data interpretation, preparation of illustrations and drafted the manuscript; JMK, JJG, MM, GP, AT, FMM, and NM helped in the interpretation of data and revised the manuscript critically for important intellectual content and for the language; PAA conceived the study, drafted the manuscript and provided overall supervision in the project; all authors read and approved the final manuscript.
